# Supplementary material for: Controlling for the species-area effect supports constrained long-term Mesozoic terrestrial vertebrate diversification
Source: Nat Commun. 2017 May 22;8:15381. doi: 10.1038/ncomms15381 (PMC5458146; doi:10.1038/ncomms15381)
Supplement: Supplementary Information — Supplementary Figures, Supplementary Tables, Supplementary Methods and Supplementary References [file ncomms15381-s1.pdf]

Supplementary Methods

Comparison of spatial metrics used to quantify geographic sampling in the fossil record. A number of summary statistics have been employed to quantify the geographic range, coverage or breadth of fossil locality data available to palaeobiologists (=palaeogeographic spread). Commonly-used measures of palaeogeographic spread include (1) convex-hull areas; (2) counts of occupied grid cells; (3) maximum great-circle distances (GCD); (4) mean or median pairwise great-circle distances; and (5) summed minimum spanning tree (MST) length. Maximum GCD and convex-hull area are range-based metrics that strongly emphasise spatial outliers and capture little-to-no information about spatial coverage, while mean or median pairwise-distances are measures of central tendency that emphasise the dispersion of points, and occupancy-based metrics record spatial coverage at the expense of either dispersion or range. Another promising variance-based metric, commonly used in the geographical sciences but apparently not employed by any palaeobiological studies, is 'standard distance' (the spatial equivalent of the standard deviation)[1].

Such metrics are generally used for two related, but distinct, purposes: (1) to discern and correct for differences in the levels of spatial sampling among geographic regions, time intervals or taxonomic groups(e.g. refs[2,3,4,5]); and (2) for estimating geographic range-sizes of fossil taxa (e.g. in studies of extinction-selectivity) (e.g. refs[5,6,7,8]). These two broad objectives may favour different approaches to measuring palaeogeographic spread, because they emphasise different aspects of the spatial information: approaches that are ideal for reconstructing range-sizes may be less well-suited to quantifying spatial sampling, and vice versa. Range-based metrics are likely to be more effective for reconstructing original geographic ranges from fossil occurrence

data, and it does not matter that these metrics place little-to-no weight on the level of spatial coverage within the inferred range. By contrast, range-based metrics are likely not appropriate if we aim to quantify spatial sampling to estimate spatiotemporal patterns of species richness in deep time. In this discussion, we focus primarily on the use of spatial metrics for quantifying spatial sampling.

In order to make fair comparisons of diversity across time and space, we must standardise the size of the geographic sampling universe from which the underlying species pool is drawn. It is necessary to standardise geographic samples because of the ubiquity of the species-area effect. If beta diversity were zero, the size of the underlying taxon-pool would be static and geographic sample-size would only influence the *amount* of data drawn from an unchanging sampling universe. Increasing palaeogeographic spread would improve sampling of the underlying species pool (producing a 'species-sampling curve'), but samples could easily be standardised to equal levels of completeness using SQS, and making fair comparisons of richness estimates between focal assemblages would be straightforward. Of course, this is not the case: ubiquitous habitat heterogeneity and geographic turnover of species means that varying spatial sampling varies the scope of the sampling universe (if beta diversity is greater than zero, larger regions must contain more species than smaller sub-regions).

Unfortunately, attempts to standardise the size of the geographic sampling universe are complicated by fact that the definition of 'spatial sampling' for occurrence-based fossil data is not clear-cut. Fossil localities (=collections; sites that represent a consistently well-constrained point in time and space) represent spatial-point data, with each defined by a single palaeolatitude and palaeolongitude. Complications arise due to the uneven distribution of fossil localities at various spatial scales. Were localities evenly and continuously distributed ('hyperdispersed'),

virtually any single measure of palaeogeographic spread would be sufficient to adequately characterise differences in spatial sampling. However, localities often have very patchy distributions: they can be densely clustered or loosely dispersed, and well-sampled regions may be separated by substantial spatial discontinuities on various scales. Distributions of spatial points can thus differ in dispersion and maximum extent/range, density, homogeneity/aggregation and overall shape. The intensity of sampling at each locality may also vary considerably. Should an outlying locality that has yielded only a single specimen contribute the same amount of spread as a locality yielding thousands of fossils? Ideally, our measure of spread would capture all of these qualities—but this, of course, is impossible. These challenges are analogous to those encountered by studies of morphological disparity and morphospace occupation, in which researchers debate the merits of range-based and variance-based measures of disparity (e.g. refs[9,10]). Our chosen metric must either represent an acceptable compromise, or we must use more than one metric. We must also accept that, in practice, it is unlikely to be possible to completely standardise spatial samples of fossil localities with respect to all of these distributional characteristics.

At a minimum, we require a measure of spatial sampling that incorporates both geographic dispersion *and* coverage in order to standardise the size of the geographic sampling universe. For example, two adjacent configurations of occupied grid-cells provide a window into a different sampling universe than the same number of occupied grid-cells separated by a substantial distance. The maximum extent of sampling alone (e.g. as estimated by maximum great-circle distance or convex-hull area) is not an effective proxy for the scope of the sampling universe unless spatial coverage *within* each region is consistent between focal assemblages. The sampling universe represented by two complete but distant samples obviously differs—perhaps

profoundly—from a sample of the same range or area that is completely sampled from extremity to extremity. Metrics that solely quantify information about the range or variance of the distribution of localities omit pertinent information about the degree of *coverage* within the study region. Conversely, metrics that primarily estimate coverage (e.g. number of occupied grid-cells) are uninformative about the *extent* or *dispersion* of sites. Since both aspects of spatial sampling affect the size of the geographic sampling universe, a metric that combines information about both aspects of geographic sampling is desirable. Below, we consider the strengths and weaknesses of commonly-used metrics.

Convex-hull area. Perhaps the most commonly-used spatial sampling metric in palaeobiology is convex-hull area, the area of the Earth's surface described by a convex-hull enclosing the localities of interest (i.e., the smallest set of points that contains all points within a convex polygon). If a convex hull can be drawn around all points without artificially inflating the true area, this metric should provide a reliable *minimum* estimate of original spatial extent. Convex-hull areas may, therefore, be an effective metric for reconstructing minimum range-sizes of fossil taxa in certain circumstances. However, the metric is inherently flawed as a more general measure of spatial sampling due to its extreme sensitivity to spatial outliers. Although adding or removing spatial points within the bounds of the convex hull perimeter cannot alter the area, changing the positions of single outliers can dramatically modify the enclosed area. In better-studied regions or intervals, the probability of sampling outlying localities increases; thus, the metric is, like any range-based statistic, highly sensitive to sample-size (indeed, more so than maximum GCD or summed MST length). Furthermore, convex hull area is downward-biased relative to other metrics if localities are arranged along a narrow transect. This is clearly problematic if convex-hull area is used as a proxy for the size of the geographic sampling

universe, as it is the *distance* between samples that dictates dissimilarity of community composition.

Occupied grid-cells. Another common approach is to tally the number of grid cells (either equal-area[5] or at regular intervals of latitude and longitude[11,12,3]) containing fossil localities. This metric provides a direct and informative indication of the degree of coverage of spatial sampling. However, it provides little direct indication of the range or dispersion of localities: grid cells may be arranged in dense clusters, long transects, or widely dispersed. It is this latter possibility that renders this metric potentially problematic when used as the sole measure of palaeogeographic spread—particularly as a proxy for sampling effects arising from the species-area relationship. If beta diversity is non-zero, a distant cell is more likely to yield different taxa and thus expand the scope of the geographic sampling universe—yet this is not reflected in the metric. Grid-cell sizes must also be chosen carefully with respect to the density and distribution of localities in order to make informative comparisons between samples. Lastly, occupied grid-cells are affected by the 'boundary problem'[1] that affects spatial-point patterns: depending on the locations of grid boundaries, multiple spatial points may be encompassed by single cell, or they may be placed into as many as four cells.

Maximum great-circle distance (max GCD). The maximum great-circle distance is the shortest distance between two points measured along the Earth's surface. Like convex-hull area, maximum GCD is a range-based measure, quantifying the maximum extent of the distribution of fossil localities. Although maximum GCD is insensitive to sample-size between the outermost points, it is inherently sensitive to the positions of outliers (albeit less so than convex hull area, which suffers the multiplicative effect of having two dimensions), and the likelihood of geographic outliers increases with sample-size. Maximum GCD would therefore provide the

same measure of palaeogeographic spread for samples containing two localities 100 km apart, samples containing an arbitrary number of localities arranged along a transect 100 km long, or an arbitrarily dense configuration of localities arranged in a circle 100 km in diameter—all of which represent substantially differently-sized geographic sampling universes. Although the consistency of maximum GCD in these circumstances may be ideal for reconstructing range-sizes of taxa from fossil data, the metric has obvious shortcomings as a measure of palaeogeographic spread that is informative about the size of the geographic sampling universe more generally.

Average pairwise great-circle distances. A less-commonly-used family of metrics involves the calculation of average (either mean[5] or median[3]) pairwise great-circle distances between localities, which reflect a combination of area and dispersion[3]. These approaches are less sensitive to outliers than simple maximum GCD, particularly if spatial points are subsampled (e.g. ref[5]). However, an even better approach can be found in the literature on descriptive spatial statistics in geography: the 'standard distance'—the equivalent of the standard deviation for spatial data. Standard distance describes the radius of a circle enclosing one standard deviation of the dataset of spatial points from the centroid point[1]. None of these methods are directly sensitive to sample size.

Minimum spanning tree (MST) length. Minimum spanning trees are constructed by finding the minimum total length of segments that can connect all nodes or spatial points. Summed MST length is a useful metric for quantifying palaeogeographic spread as it incorporates signals of range, dispersion and coverage. MSTs are also useful for algorithms that subsample spatial points, because they allow distance between clusters of points to be quantified and ranked (thus allowing natural spatial clusters to be identified). Because each additional node added to the tree

contributes some additional length unless it falls along a segment connecting two other nodes, summed MST length may be partly sensitive to sample-size. However, the extent to which sample size contributes to total MST length can be diminished by binning spatial points in equal-area grid-cells, as this partly standardises the number of nodes that may be included in an MST. A set of spatial points may also be arranged in configurations that produce the same summed MST length but different maximum extent or coverage (e.g. as quantified by maximum GCD, convex hull area or counts of occupied grid-cells). However, we feel that if certain quality criteria are applied (e.g. limiting the maximum proportional contribution the longest branch can make to a summed MST length) they are an ideal compromise if a univariate metric is desired.

What metric is most suitable for quantifying the degree of spatial sampling in the fossil record? In the present study, we aim to standardise samples of fossil localities to ensure that they represent comparable geographic sampling universes. No single metric can fully quantify all idiosyncrasies associated with the spatial distribution of fossil occurrence data and, as we have shown, each metric emphasises potentially useful information about the scope of the geographic sampling universe. The competing demands placed on an ideal palaeogeographic-spread metric is reflected in the performance of max GCD for quantifying total extent, on the one hand, and number of occupied grid-cells for quantifying fine-grained spatial coverage, on the other. Reasoning from first principals suggested that summed MST length was an acceptable compromise. To more rigorously evaluate this decision, however, we performed analyses to determine which metric was most broadly informative about the size of the geographic sampling universe. These analyses demonstrate: (1) how summed MST lengths relate to other, more commonly-used spatial sampling metrics; and (2) that the uneven distributions of localities within spatial subsamples are randomly distributed through time.

We calculated palaeogeographic spread for each interval at the regional level using a range of alternative metrics (convex-hull area, maximum GCD, counts of occupied 1° grid-cells, mean and median pairwise GCD and standard distance deviation; Supplementary Figure 1). Pairwise bivariate plots (all spatial variables logged to achieve normality) show that summed MST length is tightly correlated with all other metrics, particularly convex-hull area (Pearson's r = 0.91), maximum GCD (r = 0.97) and standard distance deviation (r = 0.91). Moreover, summed MST length exhibits the tightest correlation with the number of occupied grid-cells (r = 0.71), demonstrating that the metric represents the best compromise between other metrics, and captures a combined signal of spatial coverage, dispersion and total extent. These strong correlations between metrics can also be seen in a time-series context in Supplementary Figure 2.

We wished to determine if systematic variation in the distribution of localities within spatial subsamples (e.g. clustering or spatial discontinuities) might bias our analyses. It is challenging to compare empirical distributions of spatial points to the null expectation for how samples should be distributed if there was no bias (i.e., if points were randomly distributed according to a homogeneous Poisson process) using standard point-pattern statistics[1,13] due to boundary issues and edge-effects. This is because spatial samples must be either overlain by quadrats and a value chosen for the total area within which sampling occurs, or use a nearest-neighbour index, which is also extremely sensitive to the value chosen for total sampling area. To address this issue, we devised two novel metrics to quantify the degree of heterogeneity in the distribution of localities within spatial subsamples: (a) the proportional contribution of the longest branch in the MST, and (b) the coefficient of variation of the branch lengths within each MST. Plotting these metrics for each spatial subsample against time demonstrates no discernible temporal pattern in the distribution of intra-sample spatial heterogeneity (Supplementary Figure

7). Furthermore, we note that standardising palaeogeographic spread according to other spatial metrics would be equally vulnerable to intra-sample spatial clustering of localities.

Lastly, although our additional analyses clearly support the use of summed MST length as a palaeogeographic spread metric, we wish to acknowledge the potential for mismatch between MST length and other spatial metrics if the distribution of localities within a sample substantially differs. Binning localities within grid-cells considerably reduces these potential problems, as this procedure limits the contribution that large, densely-packed aggregations of localities can make to total spread. Furthermore, this argument against summed MST length implicitly rests on the primacy of some other spatial sampling metric (e.g. the total area described by a convex hull enveloping the outlying localities, or the maximum great-circle distance between pairs of localities). However, these purely range-based metrics are also redundant with respect to other important aspects of spatial sampling, such as the dispersion or coverage of localities within the study region (aspects that are equally important if we wish our spatial sampling metric to represent a meaningful proxy for the size of the geographic sampling universe). It would be very difficult, if not impossible, to standardise spatial samples of fossil localities with respect to all of these aspects, and we believe that summed MST length represents an appropriate compromise.

Supplementary Figures

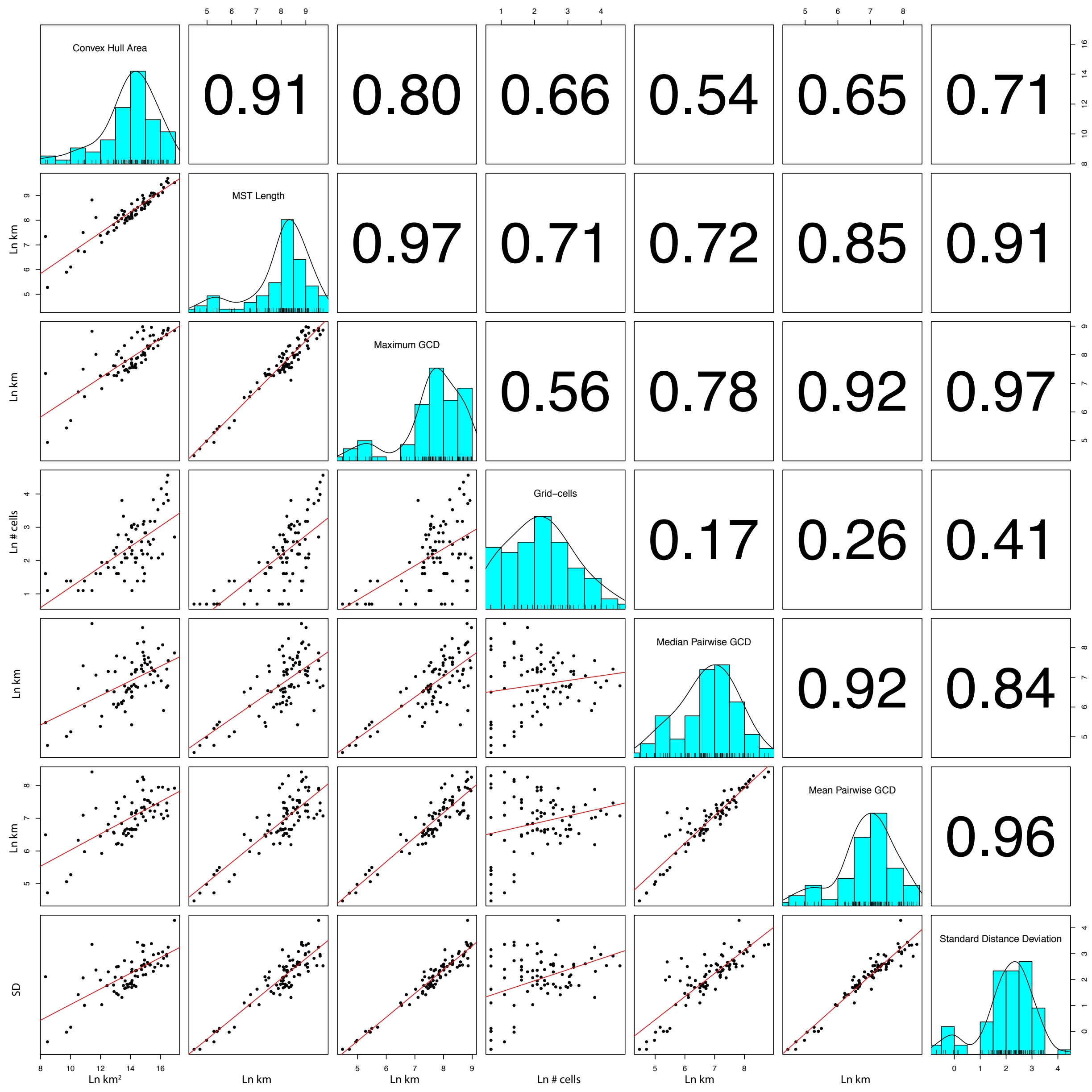

Supplementary Figure 1. Pairwise scatterplot matrix showing relationships between alternative palaeogeographic spread metrics. Data points represent regional-level palaeogeographic spreads for each interval. Numbers in upper triangle denote Pearson's *r* correlation coefficient. All variables logged to achieve normality. Red lines denote linear model fits.

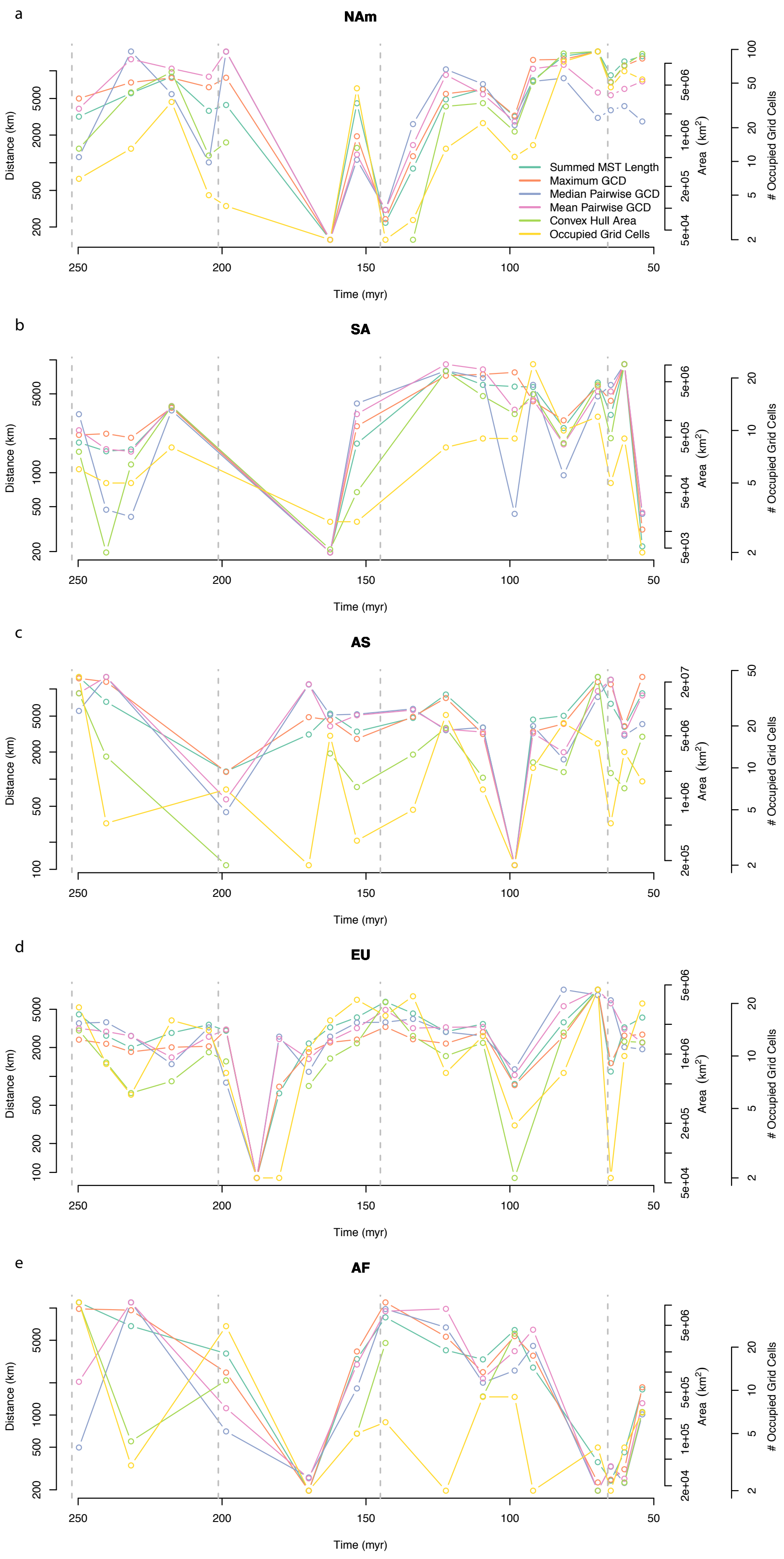

a
NAm
b
SA
c
AS
d
EU
e
AF
Distance (km)
Time (myr)
Area (km^2)
Occupied Grid Cells
Summed MST Length
Maximum GCD
Median Pairwise GCD
Mean Pairwise GCD
Convex Hull Area
Occupied Grid Cells
250
200
150
100
50

Supplementary Figure 2. Time series of regional-level palaeogeographic spreads. Palaeogeographic spread shown for summed minimum spanning tree (MST) length, maximum great-circle distance (GCD), median pairwise GCD, mean pairwise GCD, convex-hull area and counts of occupied grid-cells. (a) North America, (b) South America, (c) Asia, (d) Europe, (e) Africa.

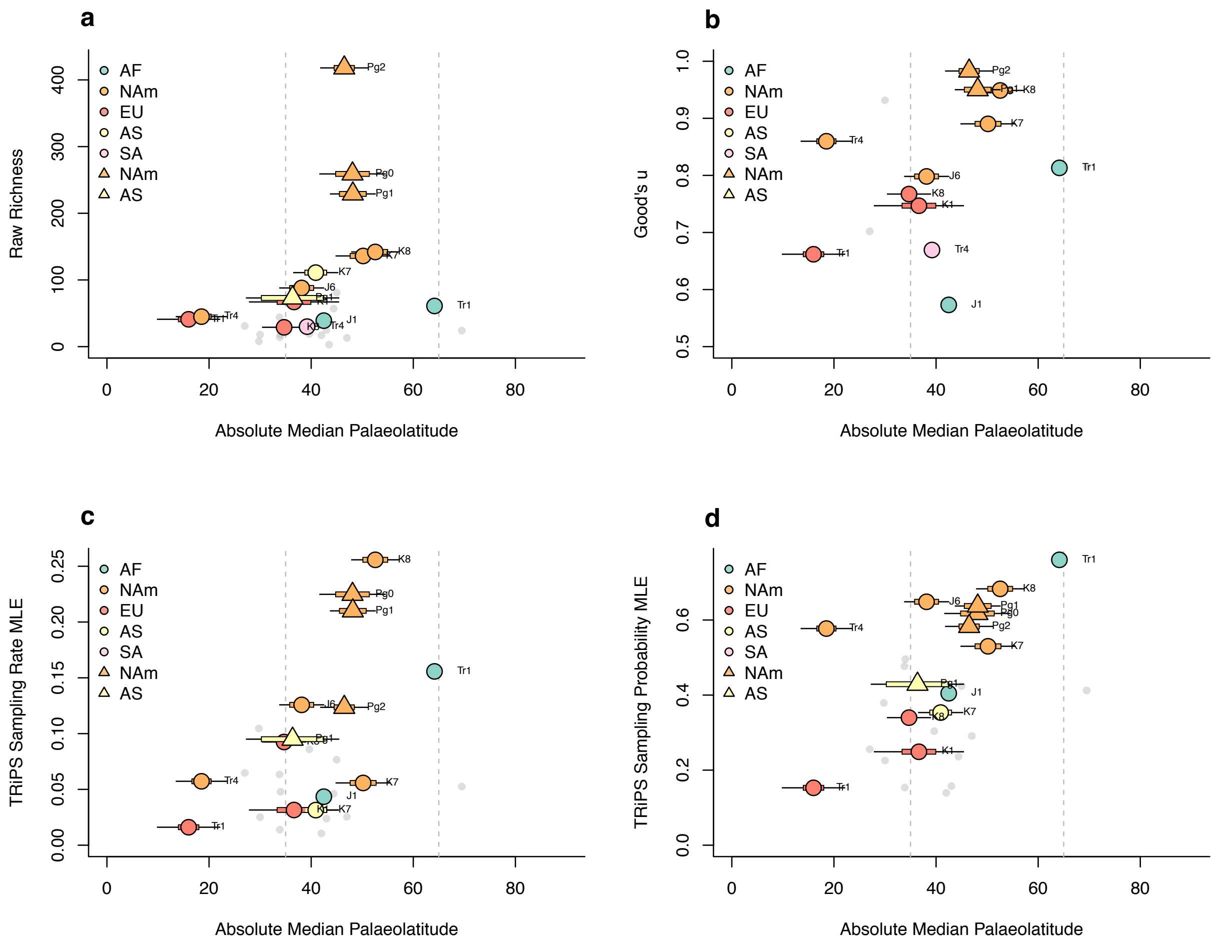

Supplementary Figure 3. Effect of spatial standardisation on palaeolatitudinal patterns. Relationship between absolute palaeolatitude and raw species richness, sampling rate and coverage for Mesozoic-early Palaeogene non-marine, non-flying tetrapods, estimated from standardised samples of palaeogeographic spread. Data points associated with fewer than 20 references shown in grey. (a) Raw richness; (b) coverage estimator Good's *u*; (c) TRiPS sampling rate; (d) TRiPS sampling probability. Dashed lines delimit palaeotemperate latitudes (35º-65º palaeolatitude). Thick error bars represent palaeolatitudinal interquartile range of spatial subsample; thin lines represent total latitudinal range.

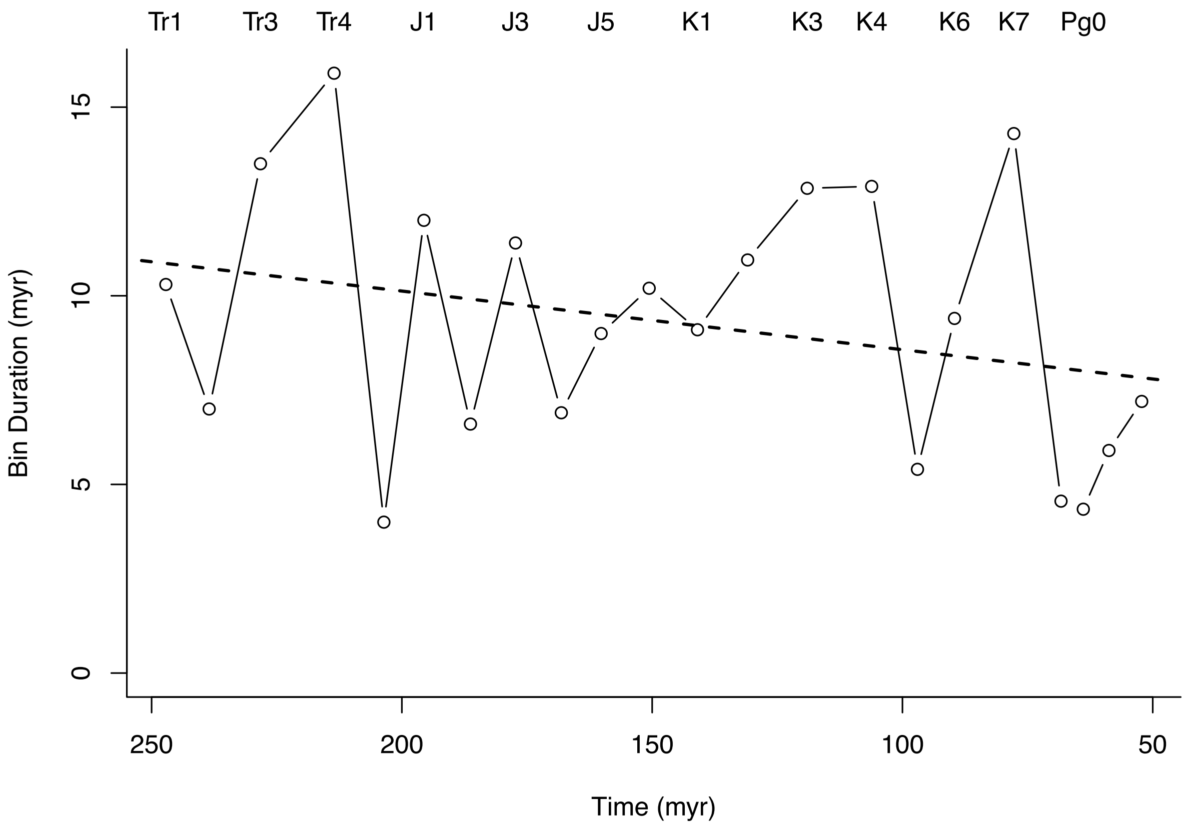

Supplementary Figure 4. Time-bin durations through time. Relationship between bin duration and time displays no statistically-significant trend**.**

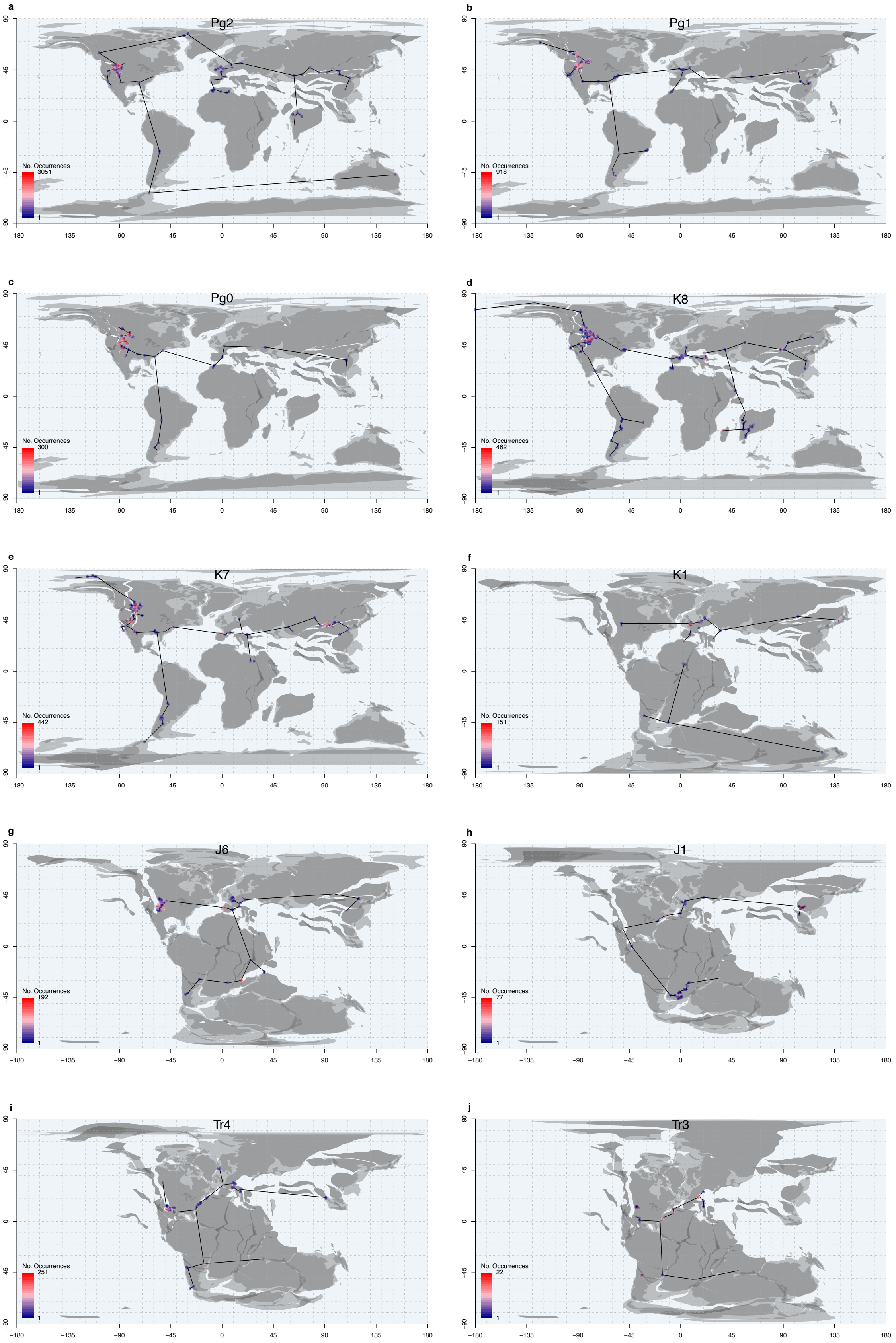
a
Pg2
No. Occurrences
3051
1
b
Pg1
No. Occurrences
918
1
c
Pg0
No. Occurrences
300
1
d
K8
No. Occurrences
462
1
e
K7
No. Occurrences
442
1
f
K1
No. Occurrences
151
1
g
J6
No. Occurrences
192
1
h
J1
No. Occurrences
77
1
i
Tr4
No. Occurrences
251
1
j
Tr3
No. Occurrences
22
1
90
45
0
−45
−90
−180
−135
−90
−45
0
45
90
135
180

Supplementary Figure 5. Global minimum spanning trees for each Mesozoic–early Paleogene time bin. Heat-map colours signify the number of occurrences known from each occupied degree grid-cell. (a) Pg2, (b) Pg1, (c) Pg0, (d) K8, (e) K7, (f) K1, (g) J6, (h) J1, (i) Tr4, (j) Tr3. Palaeomaps drawn using shapefiles from the Scotese PALEOMAP project[14].

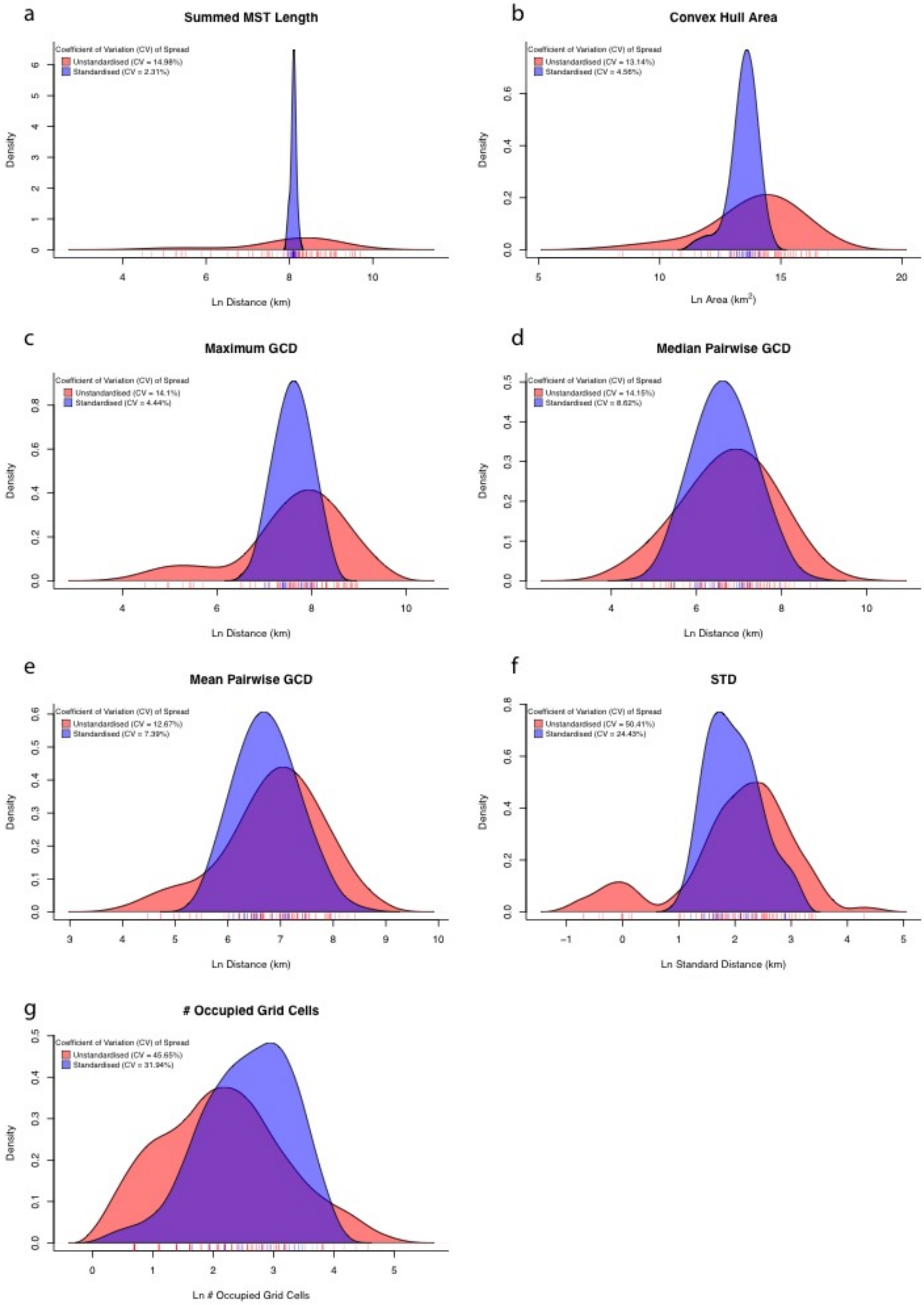
a
Summed MST Length
Coefficient of Variation (CV) of Spread
Unstandardised (CV = 14.98%)
Standardised (CV = 2.31%)
Density
Ln Distance (km)
b
Convex Hull Area
Coefficient of Variation (CV) of Spread
Unstandardised (CV = 13.14%)
Standardised (CV = 4.56%)
Density
Ln Area (km²)
c
Maximum GCD
Coefficient of Variation (CV) of Spread
Unstandardised (CV = 14.1%)
Standardised (CV = 4.44%)
Density
Ln Distance (km)
d
Median Pairwise GCD
Coefficient of Variation (CV) of Spread
Unstandardised (CV = 14.15%)
Standardised (CV = 8.62%)
Density
Ln Distance (km)
e
Mean Pairwise GCD
Coefficient of Variation (CV) of Spread
Unstandardised (CV = 12.67%)
Standardised (CV = 7.39%)
Density
Ln Distance (km)
f
STD
Coefficient of Variation (CV) of Spread
Unstandardised (CV = 50.41%)
Standardised (CV = 24.43%)
Density
Ln Standard Distance (km)
g
Occupied Grid Cells
Coefficient of Variation (CV) of Spread
Unstandardised (CV = 45.65%)
Standardised (CV = 31.94%)
Density
Ln # Occupied Grid Cells

Supplementary Figure 6. Reduction in variance following spatial standardisation.

Reduction in variance of spatial sampling resulting from MST subsampling procedure, quantified using alternative palaeogeographic spread metrics. Distributions visualised using kernel-density estimates and rug-plots. Variances before and after spatial standardisation procedure quantified using the coefficient of variation (CV %) to allow direct comparison of different spatial units. (a) summed MST length; (b) convex-hull area; (c) maximum GCD; (d) median pairwise GCD; (e) mean pairwise GCD; (f) standard distance; (g) number of occupied grid-cells.

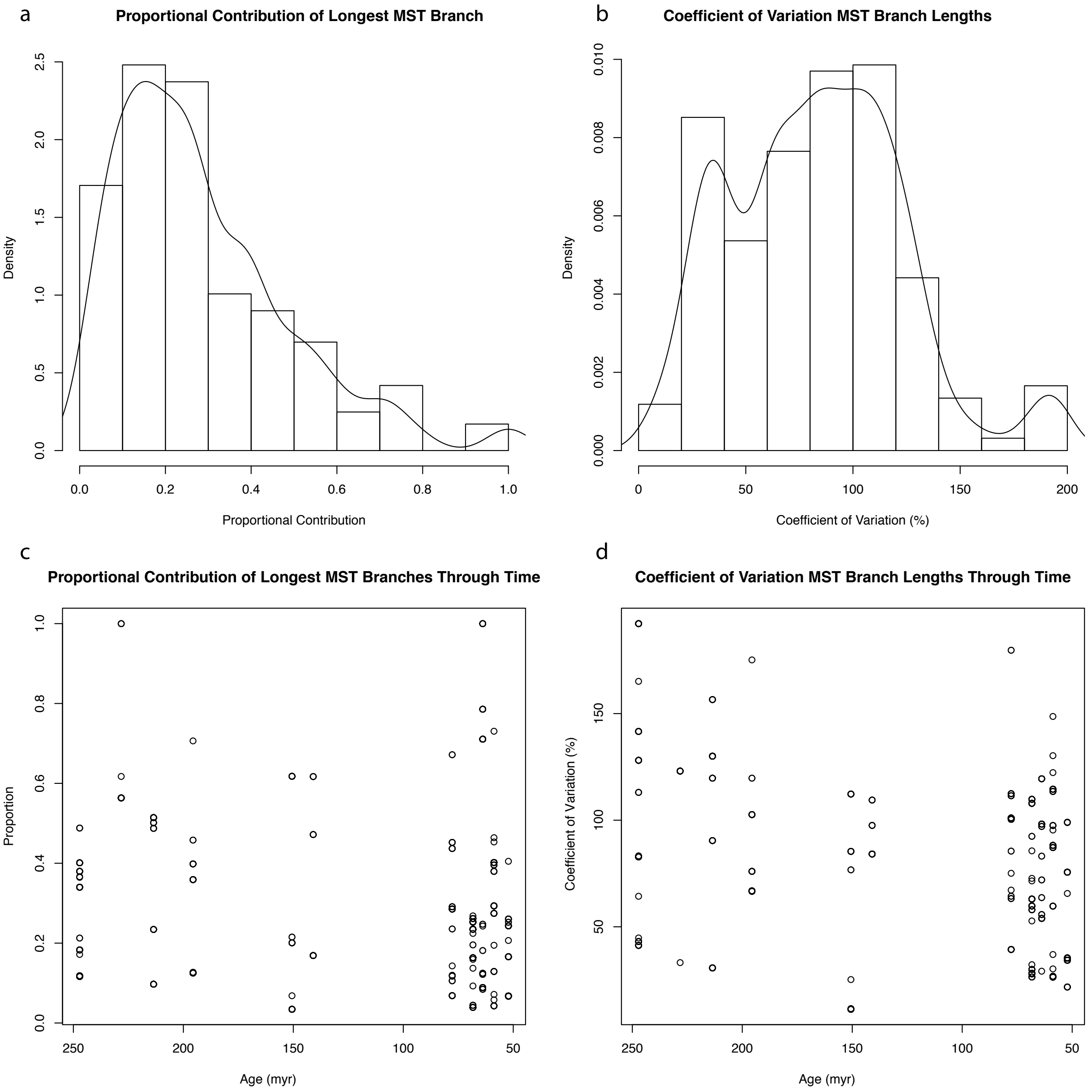

Supplementary Figure 7. Point-pattern heterogeneity within spatially-standardised subsamples, quantified by (a,c) the proportional contribution of the longest MST branch and (b,d) the coefficient of variation (CV) of the MST branch-lengths.

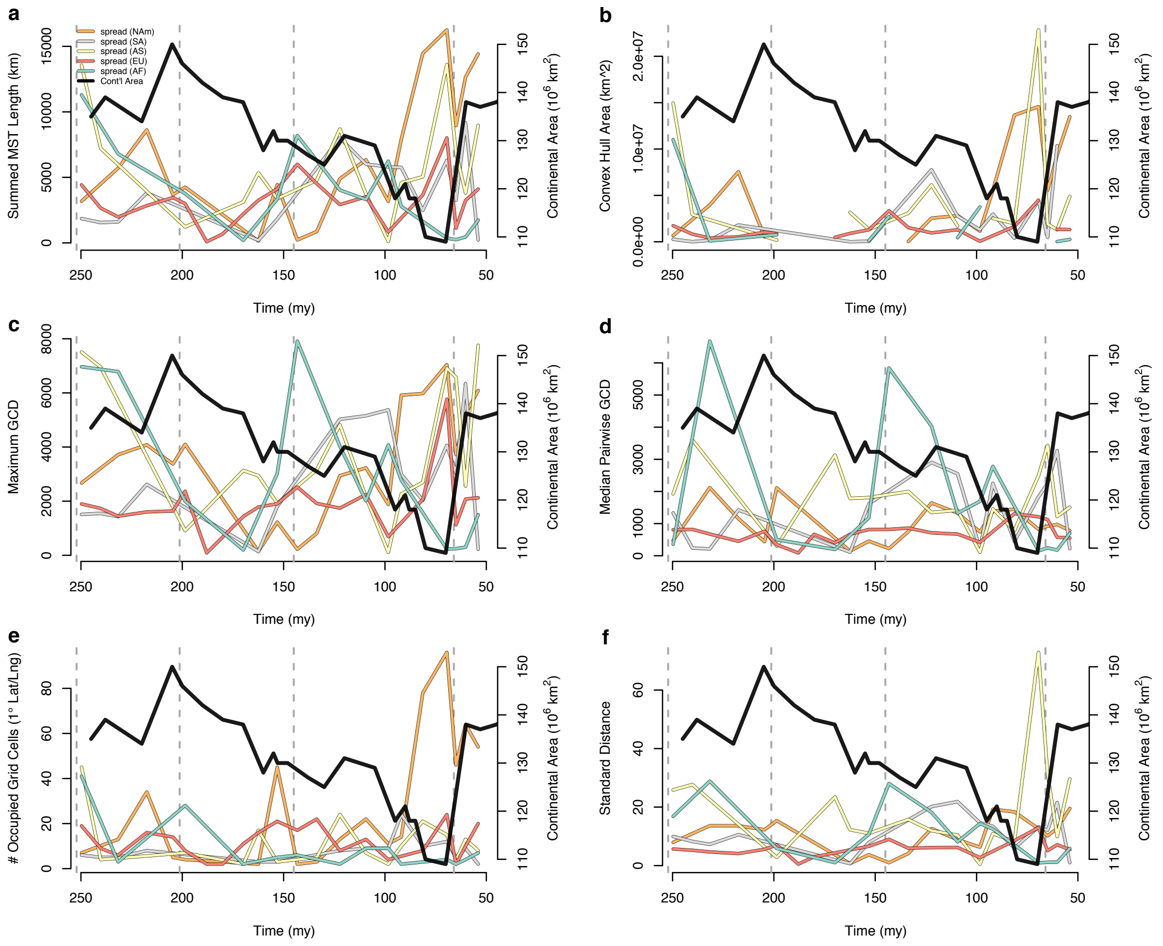

Supplementary Figure 8. Regional palaeogeographic spread and continental land-area. Relationship between regional-level palaeogeographic spread (all data points) and continental land area through time for a range of palaeogeographic spread metrics. Continental land-area values derived from ref[15]. Colours distinguish continental regions, which are defined in Supplementary Table 1.

Supplementary Tables

Supplementary Table 1: Countries included in continental regions.

Region	Countries Included
North America	United States, Canada, Mexico.
South America	Argentina, Chile, Brazil, Bolivia, Colombia, Uruguay, Peru.
Asia	China, Mongolia, South Korea, Russian Federation, North Korea.
Europe	United Kingdom, France, Germany, Italy, Switzerland, Spain, Belgium, Germany, Romania, Sweden, Czech Republic, Denmark, Slovenia, Norway, Luxembourg, Netherlands, Ukraine, Hungary, Austria, Poland, Croatia, Portugal.
Africa	Zambia, Namibia, Zimbabwe, Mali, Angola, Ethiopia, Cameroon, Malawi, Senegal, Tanzania, Eritrea, Sudan, Kenya, Libya, Niger, Tunisia, Algeria, Lesotho, Morocco, South Africa.

Supplementary Table 2: Coefficient of variation (CV) in palaeogeographic spread before and after spatial subsampling procedure.

Spatial Metric	CV (Unstandardised Spread)	CV (Standardised Spread)	Variance Reduction Factor
Convex Hull	13.1	4.6	2.9
Summed MST Length	15	2.3	6.5
Grid Cell Occupancy	45.6	31.9	1.4
Maximum GCD	14.1	4.4	3.2
Median Pairwise GCD	14.2	8.6	1.6
Mean Pairwise GCD	12.7	7.4	1.7
Standard Distance	50.4	24.4	2.1

Supplementary References

1. Burt, J. E., Barber, G. M. & Rigby, D. L. *Elementary Statistics for Geographers*. (Guilford Press, 2009).
2. Benson, R. B. J. *et al.* Near-Stasis in the Long-Term Diversification of Mesozoic Tetrapods. *PLoS Biol.* **14,** e1002359 (2016).
3. Alroy, J. Geographical, environmental and intrinsic biotic controls on Phanerozoic marine diversification. *Palaeontology* **53,** 1211–1235 (2010).
4. Barnosky, A. D., Carrasco, M. A. & Davis, E. B. The Impact of the Species–Area Relationship on Estimates of Paleodiversity. *PLoS Biol.* **3,** e266 (2005).
5. Dunhill, A. M. & Wills, M. A. Geographic range did not confer resilience to extinction in terrestrial vertebrates at the end-Triassic crisis. *Nat. Commun.* **6,** 7980 (2015).
5. Vilhena, D. A. & Smith, A. B. Spatial Bias in the Marine Fossil Record. *PLoS ONE* **8,** e74470 (2013).
6. Foote, M. & Miller, A. I. Determinants of early survival in marine animal genera. *Paleobiology* **39,** 171–192 (2016).
7. Clapham, M. E., Fraiser, M. L., Marenco, P. J. & Shen, S.-Z. Taxonomic composition and environmental distribution of post-extinction rhynchonelliform brachiopod faunas: Constraints on short-term survival and the role of anoxia in the end-Permian mass extinction. *Palaeogeogr. Palaeoclimatol. Palaeoecol.* **374,** 29–32 (2013).
8. Hopkins, M. J. How species longevity, intraspecific morphological variation, and geographic range size are related: a comparison using Late Cambrian trilobites. *Evolution* **65**, 3253-3273 (2011).
9. Ciampaglio, C. N. *et al.* Detecting changes in morphospace occupation patterns in the fossil record: characterization and analysis of measures of disparity. *Paleobiology* **27,** 695–715 (2001).
10. Wills, M. A. in *Fossils, phylogeny, and form: an analytical approach* (eds. Adrain, J. M., Edgecombe, G. D. & Lieberman, B. S.) 55–143 (Kluwer Academic Publishers, 2001).
11. Alroy, J. The shifting balance of diversity among major marine animal groups. *Science* **33**, 1191–1194 (2010).
12. Alroy, J. *et al.* Phanerozoic trends in the global diversity of marine invertebrates. *Science* **321**, 97–100 (2008).
13. Wiegand, T. & Moloney, K. A. *Handbook of Spatial Point-Pattern Analysis in Ecology*. (CRC Press, 2013).
14. Scotese, C. R. *PALEOMAP Paleoatlas for GPlates and the PaleoData Plotter Program*, http://www.earthbyte.org/paleomap-paleoatlas-for-gplates/ (2016).
15. Smith, A. G., Smith, D. G. & Funnell, B. M. *Atlas of Cenozoic and Mesozoic coastlines*. (Cambridge University Press, 1994).
